# Development and validation of Malaysian one stop crisis center service quality instrument (OSCC-Qual) for domestic violence management

**DOI:** 10.1186/s12889-024-18034-7

**Published:** 2024-04-24

**Authors:** Keng Sheng Chew, Shirly Siew-Ling Wong, Ke Lin Siew, Vanitha Kandasamy

**Affiliations:** 1 Faculty of Medicine and Health Sciences, Universiti Malaysia Sarawak, 94300 Kota Samarahan, Sarawak, Malaysia; 2 Faculty of Economics and Business, Universiti Malaysia Sarawak, 94300 Kota Samarahan, Sarawak, Malaysia; 3 Emergency and Trauma Department, Sarawak General Hospital, 93586 Kuching, Sarawak, Malaysia

**Keywords:** Domestic violence, One stop crisis center, Service quality, Multi-sectorial coordination, Validation

## Abstract

One Stop Crisis Center (OSCC) is a multi-sectorial center aimed to provide medical, social, legal, police and shelter services to survivors of domestic violence, rape, sexual assault, sodomy and child abuse. Although OSCCs have been established for almost three decades in different parts of the world including in Malaysia, there is a lack of a validated instrument to measure the service quality rendered in OSCCs. A validated instrument known as OSCC-Qual was developed using a 5-stage approach where (1) in stage 1, group discussions were conducted among all authors to identify potential items for the instrument; (2) in stage 2, content validation was performed by 13 experts using content validity index and modified kappa; (3) in stage 3, exploratory factor analysis was performed by 141 healthcare staff with experience in managing OSCC cases to validate the items as well as to identify the number of factors in the instrument; (4) in stage 4, confirmatory factor analysis was performed by 110 domestic violence survivors to ascertain the validity of the factors and items retained in stage 3 and (5) in stage 5, forward and backward translation into local Malay and Chinese languages was performed. Results: In stage 1, a total of 42 items were identified. No item was deleted in stage 2. In stage 3, a total of 7 factors (i.e., “information provision”, “competency of staff”, “professionalism”, “supportive environment”, “attitude of staff”, “multi-sectorial coordination” and “tangibles”) were identified. Four items were deleted due to poor factor loading. In stage 4, another 3 items were iteratively removed due to poor factor loading. Discriminant validity was good. Conclusion: With the availability of the 7-factor and 35-item OSCC-Qual instrument, it is hoped that the efficiency of OSCC in achieving its philosophical objectives after three decades of implementation can be unraveled and remedial actions can be taken, if necessary.

## Background

A major challenge faced by survivors of domestic violence (DV) is the onerous task of seeking services from various agencies [1]. As these agencies are often located in different places, this entails the necessity for the survivors to move from place to place. For example, the survivors may need to go to the hospital to seek treatment for their physical injuries, to the police station to make police reports and in some instances, to meet the social workers to obtain temporary shelter. To overcome these problems, an integrated center known as One Stop Crisis Center (OSCC) was first set up in Malaysia back in 1994 and subsequently established in many parts of Southeast Asia and the Western Pacific regions [1]. OSCC can be defined as “an inter-professional, health-system based center that provides survivor-centered health services alongside some combination of social, legal, police and/or shelter services to the survivors” [1].

Although OSCC has been established for almost three decades, there is a lack of literature on service quality measurement in OSCC. Unfortunately, healthcare services can be highly complex [2] and bureaucratic [3]. What is perceived as a quality service by one stakeholder might be perceived differently by another; thus, making it difficult to have a “one-size-fits-all” service quality instrument in the healthcare sector using generic service quality instruments such SERVQUAL, HEALTHQUAL, SERVPERF, PubHosQual and HospitalQual [2, 4].

Colombini et al. [1, 5] constructed a five-pronged healthcare recommendations framework in the management of cases in OSCC. These five recommendations are that (1) healthcare providers need to have good knowledge and awareness about domestic violence, protocols and referral networks to manage these cases; (2) healthcare providers need to have the skills and competency to examine and manage injuries sustained by the survivors; (3) healthcare providers need to have the right attitudes (e.g., non-judgmental and non-condescending attitude in accepting survivors as who they are and demonstrating empathy); (4) the need to have a conducive healthcare environment (i.e., sufficient time allowed for proper enquiry and response) and (5) healthcare providers need integrity and good ethical principles (e.g., keeping confidentiality, maintaining privacy, being respectful and prioritizing the survivor’s safety). Although a number of guidelines and instruments on training and integrating DV responses in health centers have been published [6–7]; to the best of our knowledge, there is a lack of instrument on measuring the quality of healthcare services rendered in the Malaysian OSCC from the perspective of DV survivors. By using Colombini et al. [5] as our overarching conceptual framework, we developed and validated a new service quality instrument to measure service quality in Malaysian OSCCs using a sequential process of item development, instrument development and instrument validation [8].

## Methodology

This study was divided into 5 stages. In stage 1, group discussions were conducted to identify potential items measuring service quality in OSCC based on Colombini et al. [5]. This was achieved through group consensus. In the event that there are discrepancies or disagreement, further discussions would be held with other emergency physicians with experience handling DV cases in OSCC in Malaysia and who were not part of this study. Apart from utilizing Colombini et al. [5], two healthcare service quality instruments that most closely reflect services in OSCC, i.e., Hong Kong Inpatient Experience Questionnaire (HKIEQ) by Wong et al. [9] as well as Rakhmawati et al. [10], were also referenced to identify the potentially relevant items. HKIEQ is a service quality instrument that measures in-patient experience in 9 dimensions, i.e., prompt access, information provision, care and involvement in decision-making, physical and emotional needs, coordination of care, respect and privacy, environment and facilities, handling patient feedback and overall care of health professionals and quality of care [9]; whereas the service quality instrument for public health center by Rakhmawati et al. [10] measures 4 dimensions, i.e., quality of healthcare service delivery, quality of healthcare personnel, quality of administration process and the adequacy of healthcare resources.

In stage 2, content validation was conducted using content validity index (CVI) and modified kappa [11, 12] to determine the relevance and appropriateness of an item. CVI is defined as the proportion of content experts who rate the relevance of an item with scores of 3 or 4 on a 4-point Likert scale (where 1 = not relevant at all, 2 = somewhat relevant, 3 = quite relevant, and 4 = highly relevant) [11]. A CVI value of 0.85 and above was considered as valid (Lynn 1986). However, to account for the possibility of chance agreement in CVI, modified kappa (κ) was analyzed (with the criteria that if κ = 0.40–0.59, the inter-rater agreement is interpreted as “fair”; if κ = 0.60–0.74, it is interpreted as “good” and if κ > 0.74, it is interpreted as “excellent”) [12]. Based on the recommendations by Lynn [11] a minimum of ten experts were required. In this study, 13 experts who had experience in managing OSCC cases in Sarawak General Hospital participated in this stage. These experts consisted of one senior consultant and head of the emergency and trauma department of Sarawak General Hospital, 9 emergency physicians, 1 nursing matron and 2 nursing sisters evaluated the relevance of items measuring service quality in OSCC.

In stage 3, exploratory factor analysis using IBM SPSS Statistics software version 25 for Mac was conducted to identify the number of factors or constructs to be extracted as well as items with good validity. Principal component analysis with varimax rotation was used as the extraction method. Scree plotting was performed with eigenvalue of > 1.0 used as the cut-off value to determine the numbers of factors. Factor loading > 0.4 was used as the criteria to determine whether an item is to be included or removed [13]. With regards to the sample size needed for this stage, the guideline for subject-to-item ratios by Costello & Osborne [14] was used. Most of the studies described in Costello & Osborne [14] used subject-to-item ratios that ranged from 3:1 to 10:1. In this case, due to a shortage of healthcare staff in the emergency department of Sarawak General Hospital, a 3:1 subject-to-item ratio was adopted, by recruiting 141 healthcare staff (medical doctors, assistant medical officers, and staff nurses) who had at least 2 years’ experience handling OSCC cases. These participants were recruited through convenience sampling. Participants then evaluated the items on a written questionnaire using a five-point Likert scale, where “1 = strongly disagree” to “5 = strongly agree”. House officers or interns as well as healthcare staff with less than 2 years’ experience in handling with OSCC cases were excluded. The reason why healthcare providers were selected as participants for stages 2 and 3 rather than patients (in this case, DV survivors) is because patients often lack the necessary knowledge to reliably list and evaluate the technical aspects of healthcare services quality, such as judging a doctor’s skills.

In stage 4, confirmatory factor analysis was conducted to further ascertain the validity of the items and factors or constructs retained from the previous stage. The measurement modelling of Partial Least Square Structural Equation Modelling using SmartPLS version 3.0 [15] was used in this stage. Cronbach alpha, composite reliability index and the rho A (ρA) coefficient (Dijkstra-Henseler’s rho) [16] were performed to determine internal consistency. For convergent validity, item factor loadings and the Average Variance Extracted (AVE) values of the constructs were determined. AVE refers to the grand mean value of the squared loadings of all items associated with a factor. Factor loading of > 0.7 is considered as acceptable for inclusion, whereas factor loading of < 0.4 would be removed [17]. For item with factor loading between 0.4 and 0.7, AVE would then be considered. If the AVE > 0.5, the item would be included [17]. For discriminant validity, the Fornell and Larcker criterion [18] and potential cross loading were evaluated. Fornell and Larcker criterion measures the degree to which an item loads higher on its own construct or factor (as measured using the square root of its AVE value) in relation to its correlation with other constructs or factors (as measured using the square of correlation values) [18]. In this stage, DV survivors who were managed in OSCC in Sarawak General Hospital were recruited as our participants. Using the F-test in G*Power software version 3.1.9.3 for Mac, with the criteria to achieve a minimum effect size of f^2^ = 0.15; α error probability = 0.05, power level (1-β) = 0.8 and with 7 independent factors, 110 participants were recruited (comprising of 92 female and 18 male participants with age ranged from 18 to 70 years old, mean = 36.8 years old). Survivors with severe injuries or those with hemodynamic instability requiring urgent medical intervention were excluded. The participants rated the items using written questionnaire with a five-point Likert scale, where “1 = strongly disagree” to “5 = strongly agree”.

In stage 5, translation into local languages that are familiar to the Malaysian communities were conducted. The translation processes outlined by Beaton et al. [19], i.e., (1) forward translation (2) synthesis and harmonization of forward translated versions (3) back translation and (4) review and resolution of discrepancies (stage 5). Professional translation service was utilized for the forward and backward translation.

The medical research ethics approval from the Malaysian Medical Research and Ethics Committee (NMRR-20-1437-5483; https://nmrr.gov.my/) was obtained before starting this study. No personal identifiable information such as the participants’ names, national identity number or passport number, etc. were collected in this study. All participants in stages 2 to 4 were assured that their data were used solely for the purpose of this study and not for other purposes.

## Results

In stage 1, which focused on the identification of potentially relevant items, a total of 42 items were considered for inclusion in our instrument. An item qualified for inclusion if it reflects OSCC services described in Colombini et al. [5].

**Fig. 1:**
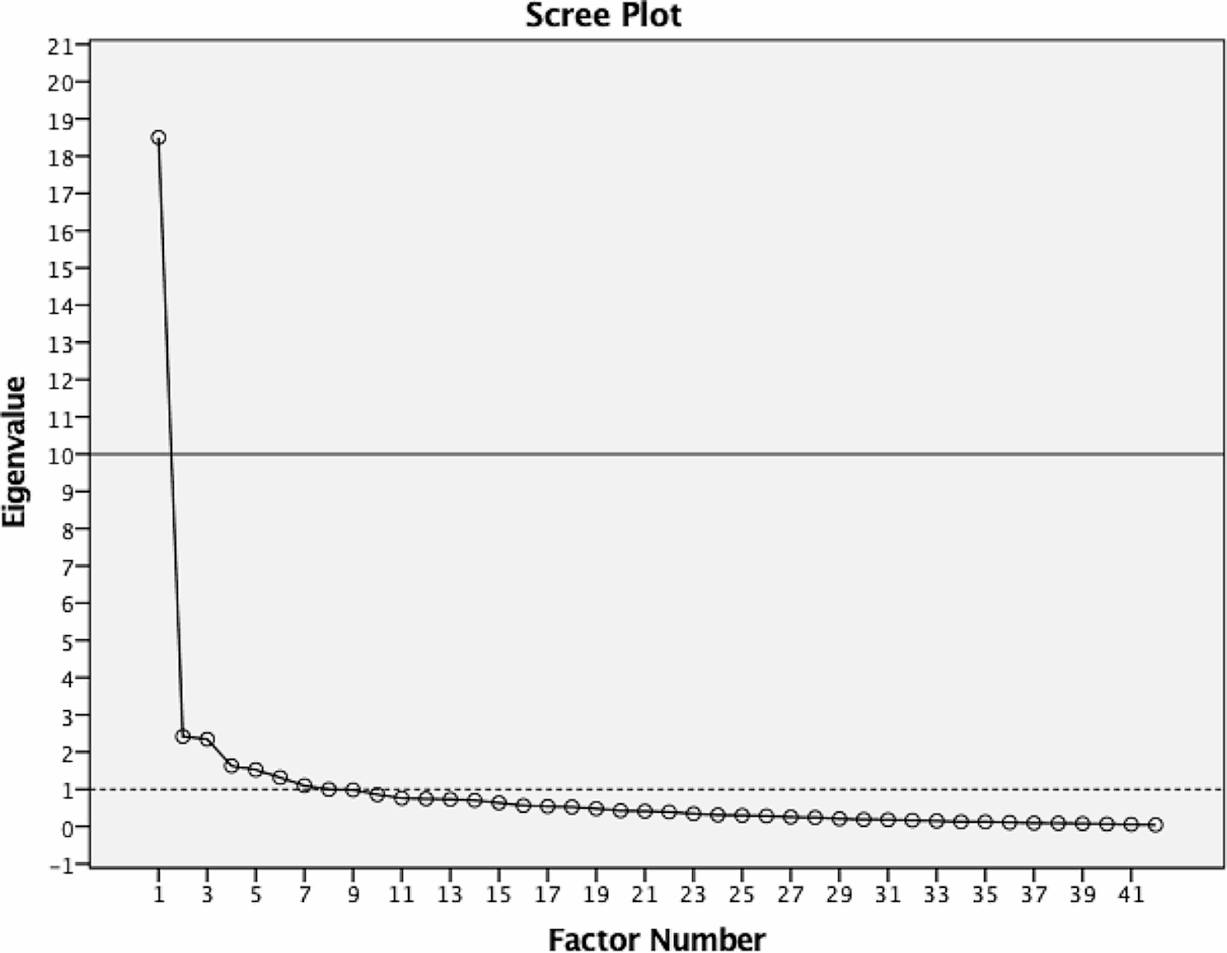
Scree plot performed in stage 3 showing 7 factors with eigenvalue > 1.0

In stage 2, which focused on content validation, the relevance and suitability of each item were assessed using content validation index (CVI) result and modified kappa (κ). Based on these analyses, all 42 items were retained (see Table 1). In stage 3, which focused on identifying the number of factors and items (from the previous stage) using exploratory factor analysis, 7 distinct factors or constructs encompassing 38 items were identified. Four items, i.e., “The cleanliness of the OSCC room is good”, “The cleanliness of toilet/bathroom in OSCC room is good”, “I receive information about the results of the treatment/procedure after its implementation”, and “The healthcare staff involves me in decision making related to my OSCC case management”, were deleted due to poor factor loadings of < 0.4 (refer to the scree plot of factors with eigenvalue > 1.0 in Fig. 1). Based on the items loaded to these factors, the factors were named as (1) “information provision” (2) “competency of staff” (3) “professionalism” (4) “supportive environment” (5) “attitude of staff” (6) “multi-sectorial coordination” and (7) “tangibles”.

**Table 1:** Content validation Index (CVI) analysis in stage 2

Item	N	A	Pc	CVI	Modified kappa (κ)	Interpretation of Modified kappa (κ)
The time taken to admit me to OSCC when I first arrive is fast	13	13	0.00012	1.000	1.00	Excellent
I am bothered by the surrounding noise in OSCC	13	11	0.00952	0.846	0.84	Excellent
The cleanliness of OSCC room is good	13	13	0.00012	1.000	1.00	Excellent
The cleanliness of toilet/bathroom in OSCC room is good	13	13	0.00012	1.000	1.00	Excellent
Quality of food in OSCC is good	13	11	0.00952	0.846	0.84	Excellent
I am allowed to choose food (e.g., vegetarian, diabetic, etc.) when I am in OSCC	13	11	0.03491	0.846	0.76	Excellent
The doctors give me clear answers regarding the questions that I have	13	13	0.00012	1.000	1.00	Excellent
The nurses give me clear answers regarding the questions that I have	13	13	0.00012	1.000	1.00	Excellent
I have trust in the nurses who treat me	13	13	0.00012	1.000	1.00	Excellent
I have trust in the doctors who treat me	13	13	0.00012	1.000	1.00	Excellent
There are adequate healthcare staff on duty to care for me	13	12	0.00159	0.923	0.92	Excellent
I receive conflicting or contradicting information from different healthcare staff	13	11	0.00952	0.846	0.84	Excellent
I have enough opportunity to ask questions to the doctor/healthcare staff	13	12	0.00159	0.923	0.92	Excellent
There is space for me to discuss/vent out my worries or fears with healthcare staff	13	12	0.00159	0.923	0.92	Excellent
My privacy is maintained when discussing condition/treatment/procedure	13	12	0.00159	0.923	0.92	Excellent
My privacy is maintained when being examined or treated	13	12	0.00159	0.923	0.92	Excellent
The time taken by healthcare staff in answering my call is fast	13	11	0.00952	0.846	0.84	Excellent
I receive enough help from healthcare staff	13	12	0.00159	0.923	0.92	Excellent
I receive information about the results of the treatment/procedure after its implementation	13	13	0.00012	1.000	1.00	Excellent
The healthcare staff involves me in decision making pertaining to the management of my case in OSCC	13	13	0.00012	1.000	1.00	Excellent
The time taken for the admission/discharge/transfer from OSCC is fast (if applicable)	13	13	0.00012	1.000	1.00	Excellent
I receive clear explanation about the purpose of treatment/procedure	13	13	0.00012	1.000	1.00	Excellent
I receive clear explanation about the condition or injuries sustained	13	13	0.00012	1.000	1.00	Excellent
I receive clear explanation about the side effects/complication of the treatment	13	13	0.00012	1.000	1.00	Excellent
I receive information about the danger or warning signals to watch out for after discharge	13	12	0.00159	0.923	0.92	Excellent
I am well informed on who to contact after discharge from OSCC in case of any emergency	13	13	0.00012	1.000	1.00	Excellent
The information I received from the healthcare staff is useful	13	13	0.00012	1.000	1.00	Excellent
I am treated with respect and dignity in OSCC	13	13	0.00012	1.000	1.00	Excellent
There is space to complain about the care received in OSCC	13	13	0.00012	1.000	1.00	Excellent
The care that I received in OSCC matches my expectations	13	13	0.00012	1.000	1.00	Excellent
The doctors are competent in managing my condition	13	13	0.00012	1.000	1.00	Excellent
The nurses are competent in managing my condition	13	13	0.00012	1.000	1.00	Excellent
The healthcare staff are hospitable and courteous	13	13	0.00012	1.000	1.00	Excellent
The healthcare staff are sincere in helping me	13	13	0.00012	1.000	1.00	Excellent
The healthcare staff are willing to listen to my problems/complaints	13	13	0.00012	1.000	1.00	Excellent
The healthcare staff are able to understand/comfort my worries/fears	13	13	0.00012	1.000	1.00	Excellent
The healthcare staff constantly interrupt me before I finish talking	13	11	0.00952	0.846	0.84	Excellent
The registration procedure in OSCC is easy	13	13	0.00012	1.000	1.00	Excellent
The registration process in OSCC is fast	13	13	0.00012	1.000	1.00	Excellent
Making police report from OSCC is easy (if relevant)	13	13	0.00012	1.000	1.00	Excellent
Making police report from OSCC is fast (if relevant)	13	13	0.00012	1.000	1.00	Excellent
The patient’s overall rating of care received in OSCC	13	12	0.00159	0.923	0.92	Excellent

**Note**:

◦ The formula for modified kappa statistic (κ) = (CVI– pc)/(1– pc), where pc represents probability of a chance occurrence

◦ P_c_ is the probability of chance of occurrence. The formula for pc is: N!/[A!*(N-A)!]*0.5^N^ where N = the number of judges, A = the number agreeing on good relevance

◦ Evaluation criteria for modified kappa (κ): κ = fair (0.40–0.59), κ = good (0.60–0.74) and κ = excellent (> 0.74)

◦ CVI should be 0.88 and above to establish validity with a *p* < 0.05

**Fig. 2:**
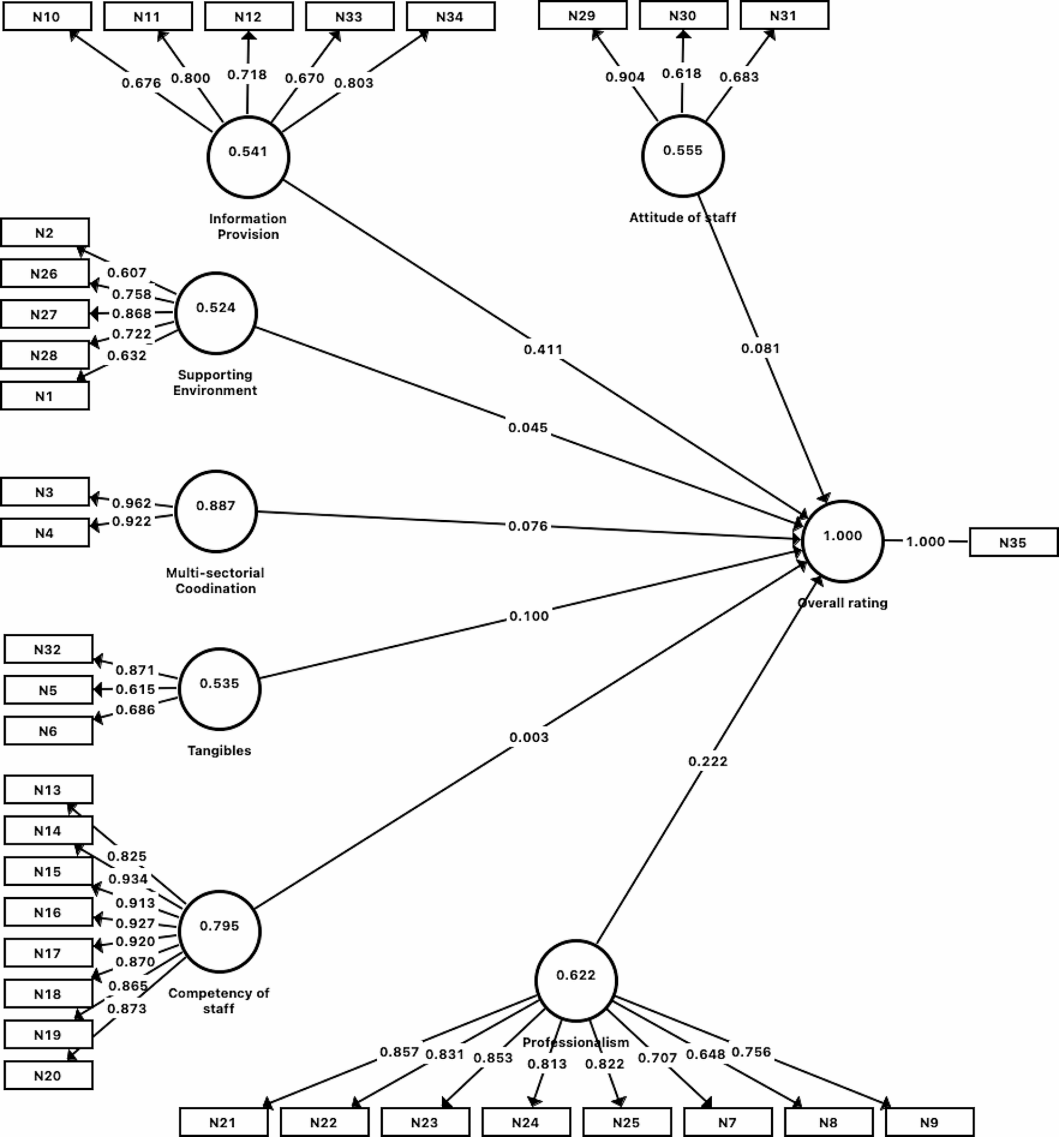
Path Analysis Model in stage 4. Note: (1) the values in the inner model in each of the factors refer to the AVE values; (2) the values in the outer model refer to the factor loadings; (3) for the descriptions of the item codes, refer the Table 3

In stage 4, which focused on further validating and ensuring the reliability of factors and items identified using confirmatory factor analysis, 3 items were iteratively removed due to poor factor loadings. Item “I am bothered by the surrounding noise in OSCC” was first removed due to factor loading of 0.222. Item “The time taken for the admission/discharge/transfer from OSCC is fast (if applicable)” was subsequently removed due to factor loading of 0.565 (between 0.4 and 0.7) and the overall AVE of 0.451 (less than 0.5) for the construct “supportive environment”. After deleting this item, the AVE for the construct “supportive environment” improved to 0.524. Item “I receive clear explanation about the side effects/complication of the treatment” was finally deleted due to factor loading of 0.534 and the overall AVE of 0.494 for the construct “information provision”. With the removal of this item, the AVE for “information provision” improved to 0.541. Discriminant validity was affirmed as evidenced by the absence of significant cross loading as well as the fulfillment of the Fornell and Larcker criterion [18]. (refer to Table 2; Fig. 2 for details).

**Table 2:** Internal Consistency Reliability and Convergent Validity for stage 4

Construct	Internal Consistency Reliability			Convergent Validity		
	Cronbach’s Alpha	Composite Reliability	Dijkstra–Henseler’s Rho (ρ)	Items	Factor Loadings	Average Variance Extracted
Supportive environment	0.774	0.844	0.807	N1	0.632	0.524
				N2	0.607	
				N26	0.758	
				N27	0.868	
				N28	0.722	
Multi-sectorial coordination	0.877	0.940	0.952	N3	0.962	0.887
				N4	0.922	
Tangibles	0.669	0.772	0.822	N5	0.615	0.535
				N6	0.686	
				N32	0.871	
Professionalism	0.912	0.929	0.914	N7	0.707	0.622
				N8	0.648	
				N9	0.756	
				N21	0.857	
				N22	0.831	
				N23	0.853	
				N24	0.813	
				N25	0.822	
Information Provision	0.789	0.854	0.814	N10	0.676	0.541
				N11	0.800	
				N12	0.718	
				N33	0.670	
				N34	0.803	
Competency of staff	0.963	0.969	0.970	N13	0.825	0.795
				N14	0.934	
				N15	0.913	
				N16	0.927	
				N17	0.920	
				N18	0.870	
				N19	0.865	
				N20	0.873	
Attitude of staff	0.718	0.785	0.997	N29	0.904	0.555
				N30	0.618	
				N31	0.683	

**Table 3:** Final version of OSCC-Qual Instrument after Confirmatory Factor Analysis

Code	Item	Items are to be rated on a scale of 5 where:				
		1 = Strongly Disagree Sangat Tidak Setuju 非常不同意	2 = Disagree Tidak Setuju 不同意	3 = Neutral Neutral 中立	4 = Agree Setuju 同意	5 = Strongly Agree Sangat Setuju 非常同意
N1	The registration process in OSCC is fast *Proses pendaftaran di OSCC adalah cepat* OSCC的登记流程很快速	1	2	3	4	5
N2	The registration procedure in OSCC is easy *Prosedur pendaftaran di OSCC adalah mudah* OSCC的登记程序很简单	1	2	3	4	5
N3	Making police report from OSCC is fast (if relevant) *Membuat laporan polis dari OSCC adalah cepat (jika berkenaan)* 在OSCC里, 我能快速地向警方报案(如相关)	1	2	3	4	5
N4	Making police report from OSCC is easy (if relevant) *Membuat laporan polis dari OSCC adalah mudah (jika berkenaan)* 在OSCC里, 我能简单地向警方报案(如相关)	1	2	3	4	5
N5	I am allowed to choose food (e.g., vegetarian, diabetic, etc.) when I am in OSCC *Saya dibenarkan memilih makanan (spt. vegetarian, diabetes dll.) semasa saya berada di OSCC* 在OSCC里, 我可以选择所需的食物(例如: 素食餐、控制糖尿病的饮食餐等等)	1	2	3	4	5
N6	Quality of food in OSCC is good *Kualiti makanan yang dihidangkan di OSCC hospital adalah baik* OSCC所提供的食物质量很好	1	2	3	4	5
N7	My privacy is maintained when being examined or treated *Privasi saya dijaga semasa diperiksa atau dirawat* 在接受检查或治疗时, 我的隐私受到维护	1	2	3	4	5
N8	My privacy is maintained when discussing condition/treatment/procedure *Privasi saya dijaga semasa perbincangan tentang keadaan/rawatan/prosedur saya* 在讨论自身的病情/治疗/医疗程序时, 我的隐私受到维护	1	2	3	4	5
N9	I am treated with respect and dignity in OSCC *Saya dilayani dengan penuh rasa hormat dan maruah di OSCC* 在OSCC接受治疗期间, 我受到尊重和有尊严的对待	1	2	3	4	5
N10	I receive clear explanation about the condition or injuries sustained *Saya mendapat penerangan yang jelas mengenai keadaan atau kecederaan yang saya alami* 对于自身状况或伤势, 我得到明确的解释	1	2	3	4	5
N11	The information I received from the healthcare staff is useful *Maklumat yang saya terima dari kakitangan kesihatan adalah berguna* 我从医疗人员处得到的信息非常有用	1	2	3	4	5
N12	I receive clear explanation about the purpose of treatment/procedure *Saya menerima penerangan yang jelas mengenai tujuan rawatan/prosedur* 关于治疗的目的/医疗程序, 我得到明确的解释	1	2	3	4	5
N13	I receive enough help from healthcare staff *Saya mendapat bantuan yang mencukupi daripada kakitangan kesihatan di sini* 我从医疗人员处得到充足的帮助	1	2	3	4	5
N14	The healthcare staff are sincere in helping me *Kakitangan kesihatan di sini membantu saya dengan ikhlas* 医疗人员真诚地帮助我	1	2	3	4	5
N15	The healthcare staff are willing to listen to my problems/complaints *Kakitangan kesihatan di sini bersedia mendengar masalah/keluhan saya* 医疗人员十分愿意倾听我所提出的问题/投诉	1	2	3	4	5
N16	The healthcare staff are hospitable and courteous *Kakitangan kesihatan di sini adalah ramah dan sopan* 医疗人员热情服务和礼貌待人	1	2	3	4	5
N17	The healthcare staff are able to understand/comfort my worries/fears *Kakitangan kesihatan di sini dapat memahami/meredakan kebimbangan/ketakutan saya* 医疗人员能够理解/安抚我的担忧/恐惧	1	2	3	4	5
N18	The care that I received in OSCC matches my expectations *Penjagaan yang saya terima di OSCC memenuhi jangkaan saya* OSCC所提供的诊疗服务与我的期望相符	1	2	3	4	5
N19	The nurses are competent in managing my condition *Jururawat yang menjaga saya adalah mahir* 护士们拥有足够的胜任能力去处理我的状况	1	2	3	4	5
N20	The doctors are competent in managing my condition *Doktor yang menjaga saya adalah mahir* 医生们拥有足够的胜任能力去处理我的状况	1	2	3	4	5
N21	The doctors give me clear answers regarding the questions that I have *Doktor di sini memberikan jawapan yang jelas tentang persoalan yang saya ada* 医生们能清晰地解答我所提出的问题	1	2	3	4	5
N22	The nurses give me clear answers regarding the questions that I have *Jururawat di sini memberikan jawapan yang jelas tentang persoalan yang saya ada* 护士们能清晰地解答我所提出的问题	1	2	3	4	5
N23	I have trust in the nurses who treat me *Saya menaruh kepercayaan kepada jururawat yang merawat saya* 我对照料我的护士们十分有信心	1	2	3	4	5
N24	I have trust in the doctors who treat me *Saya menaruh kepercayaan kepada doktor yang merawat saya* 我对治疗我的医生们十分有信心	1	2	3	4	5
N25	I have enough opportunity to ask questions to the doctor/healthcare staff *Saya mempunyai peluang yang cukup untuk mengemukakan soalan kepada doktor/kakitangan kesihatan* 我有充足的机会向医生/医疗人员提出我的疑问	1	2	3	4	5
N26	There is space for me to discuss/vent out my worries or fears with healthcare staff *Terdapat ruang untuk saya membincangkan/mengatasi kebimbangan atau ketakutan saya dengan kakitangan kesihatan* 医疗人员为我提供充足的空间, 让我向他们讨论/倾诉我的担忧或恐惧	1	2	3	4	5
N27	There are adequate healthcare staff on duty to care for me *Terdapat kakitangan kesihatan yang mencukupi untuk menjaga saya* OSCC拥有足够的值班医疗人员为我提供诊疗服务	1	2	3	4	5
N28	The time taken to admit me to OSCC when I first arrive is fast *Masa yang diambil untuk memasukkan saya ke OSCC apabila saya mula-mula tiba adalah cepat* 我初到急诊时, 接纳我进入OSCC的时间很短	1	2	3	4	5
N29	The time taken by healthcare staff in answering my call is fast *Masa yang diambil oleh kakitangan kesihatan dalam menjawab panggilan saya adalah cepat* 医疗人员迅速回应我的呼叫	1	2	3	4	5
N30	The healthcare staff constantly interrupt me before I finish talking *Kakitangan kesihatan sentiasa menganggu saya sebelum saya selesai bercakap* 在事实陈述/对话的过程中, 医疗人员不时打断我的倾诉	1	2	3	4	5
N31	I receive conflicting or contradicting information from different healthcare staff *Saya menerima maklumat yang bertentangan atau bercanggahan daripada kakitangan kesihatan yang berbeza* 我从不同医疗人员那里了解到了不一致或矛盾的信息	1	2	3	4	5
N32	There is space to complain about the care received in OSCC *Terdapat ruang untuk mengadu tentang perawatan yang diterima di OSCC* OSCC拥有让我提出对医疗服务不满的平台	1	2	3	4	5
N33	I receive information about the danger or warning signals to watch out for after discharge *Maklumat tentang bahaya atau isyarat amaran yang harus diperhatikan setelah discaj dari hospital telah diberikan kepada saya* 我得到关于出院后需要注意的危险或警报信号	1	2	3	4	5
N34	I am well informed on who to contact after discharge from OSCC in case of any emergency *Maklumat mengenai pihak yang harus saya hubungi sekiranya berlaku sebarang kecemasan selepas discaj dari hospital telah diberikan kepada saya* 我充分了解从OSCC出院后, 每当遇到任何紧急事故时, 我应该与谁联系	1	2	3	4	5
N35	My overall rating of care received in OSCC is good *Penilaian keseluruhan penjagaan saya diterima di OSCC adalah baik* 我在OSCC接受到了全面优质的诊疗服务	1	2	3	4	5

## Discussion

In a five-stage study, potential items for measuring service quality in OSCC were first identified and further refined and validated through exploratory and confirmatory factor analyses, leading to the development of a 35-item instrument across seven constructs. The final version of OSCC-Qual was translated into Malay and Chinese languages to maintain cultural relevancy. This instrument encompasses 7 dimensions i.e., (1) “information provision” (2) “competency of staff” (3) “professionalism” (4) “supportive environment” (5) “attitude of staff” (6) “multi-sectorial coordination” and (7) “tangibles”, reflecting a comprehensive evaluation of service quality in most OSCCs globally.

Unlike other healthcare services, the services rendered in OSCC must be compassionately sensitive, protective and able to facilitate multi-agency coordination in order to reduce the risk of stigmatization and re-traumatization. Stigmatization refers to the internalization of negative connotations such as shame, blame and guilt so much so that these negative beliefs become entrenched in the daily lives of the survivors and may even paralyze them psychologically [20]. Secondary victimization refers to the process of re-traumatization experienced by the survivors due to the inappropriate or insensitive comments made by staff (e.g., putting the blame on them) [21].

However, as the adage says, “you can’t manage well what you don’t measure”. To measure requires a measurement tool. In this regard, we believe that the newly developed 7-factor and 35-item OSCC-Qual instrument can capture the essence of the core services given in OSCC including items such as “My privacy is maintained when being examined or treated”, “I am treated with respect and dignity in OSCC” to capture the degree of stigmatization and secondary victimization as perceived by the survivors. Indeed, each of these factors is aligned with one of the five healthcare response recommendations [5]. For example, factors “supportive environment”, “multi-sectorial coordination” and “tangibles” are aligned with the recommendation of “the need to have a conducive healthcare environment (i.e., sufficient time allowed for proper enquiry and response)” in Colombini et al. [5]. The details of the mapping between the factors in OSCC-Qual instrument with the healthcare response recommendations is given in Table 4.

**Table 4:** Mapping of Factors in OSCC-Qual with Healthcare Responses Recommendations

Factors in OSCC-Qual	Healthcare response recommendations (Colombini et al.) [5]
Supportive environment	The need to have a conducive healthcare environment (i.e., sufficient time allowed for proper enquiry and response)”
Multi-sectorial coordination	
Tangibles	
Professionalism	Healthcare providers need integrity and good ethical principles
Information provision	Healthcare providers need to have good knowledge and awareness about domestic violence, protocols and referral network for managing domestic violence
Competency of staff	Healthcare providers need to have the skills and competency to examine and manage injuries sustained by the survivors
Attitude of staff	Healthcare providers need to have the right attitudes (e.g., non-judgmental and non-condescending attitude in accepting survivors as who they are and demonstrating empathy)

In stage 5, forward and backward translation of the instrument from English language to Malay and Chinese languages were performed. The backward translated version and the original version in English were compared by the authors who are proficient in the respective languages (i.e., KSC for Malay language version and SSLW for the Chinese language version) to ensure that the two versions conveyed the same meanings and that the semantic equivalence between the original English version and the translated versions in Malay and Chinese languages were preserved. The final version of OSCC-Qual has 35 items in 7 factors or construct (see Table 3 for the complete version together with the Malay and Chinese languages translation).

This study has several limitations that should be mentioned. First, this patient-centric instrument is designed with more positively framed items compared to than negatively framed items. Considering that patients are often considered as a vulnerable group, there is a high susceptibility to social desirability bias, i.e., the tendency to underreport attitudes believed to be perceived negatively and overreport those believed to be viewed positively during self-assessments or surveys [22]. Additionally, considering that patients tend to overrate items framed in a positive manner, there is a high susceptibility to risk of acquiescence bias, the tendency for participants to agree with the statement regardless of its contents, leading to artificially inflated rating [23]. Furthermore, many patients may not have the technical knowledge to reliably evaluate healthcare services quality. Therefore, evaluations using OSCC-Qual may not fully reflect the true service quality. Incorporating other objective and subjective metrics from various healthcare providers is vital for a holistic assessment of OSCC service quality. Third, this study was conducted on DV survivors only, which does not capture all forms of violence that patients presenting to OSCCs may experience, such as child abuse, elder abuse, conflict-related sexual violence, etc. The perception of the survivors of these traumas may differ from that of the DV survivors. Fourth, the validation process of this instrument involved only participants from the state of Sarawak or those residing in Sarawak. Hence, as this was a single center study, there may be socio-cultural forces that might have reduced generalizability of the instrument to OSCCs in other parts of the world and even within Malaysia.

Nonetheless, we believe that even with the possibility of geographical variations in OSCC setting and OSCC management in different parts of the world [1], OSCC-Qual instrument is generic enough to capture the core OSCC services, particularly hospital-based OSCC, as it is girded on the foundational principles of OSCC establishment [1, 5]. Conducting future studies in diverse settings is essential to validate and to potentially adapt this instrument and substantiating its applicability and reliability across different socio-cultural environments and service structures.

## Conclusion

In conclusion, this 7-factor and 35-item OSCC-Qual instrument was developed through a 5-stage process of item generation and development, content validation, exploratory factor analysis, confirmatory factor analysis and translation of the instrument into the Malay and Chinese languages that are familiar to most Malaysians. With the availability of this objective measurement tool, it is hoped that the answers as to whether we have successfully achieved the philosophical objectives of OSCC after three decades of implementation can soon be unraveled and perhaps, necessary remedial steps can be taken to ensure that OSCCs continue to meet the delicate needs of the survivors.

## Data Availability

The data used for exploratory factor analysis and confirmatory factor analysis are available here: https://tinyurl.com/4vn7yrc9 or by contacting the corresponding author.
